# On the genera *Qiongocera* and *Relictocera* (Araneae, Psilodercidae) from Southeast Asia

**DOI:** 10.3897/zookeys.862.33078

**Published:** 2019-07-09

**Authors:** Wan-Jin Chang, Fengyuan Li, Shuqiang Li

**Affiliations:** 1 Institute of Zoology, Chinese Academy of Sciences, Beijing 100101, China Institute of Zoology, Chinese Academy of Sciences Beijing China

**Keywords:** Cave, China, Ochyroceratidae, sexual dimorphism, Thailand, Vietnam

## Abstract

Four new species are described in two psilodercid genera, *Qiongocera* Li & Li, 2017 and *Relictocera* Li & Li, 2017: *Qiongoceraluoxuan* Li & Li, **sp. nov.** (♂♀) from China, *Relictocerawugen* Li & Li, **sp. nov.** (♂♀) and *R.sigen* Li & Li, **sp. nov.** (♂) from Vietnam, and *R.qianzi* Li & Li, **sp. nov.** (♂♀) from Thailand. These genera were previously thought to be monotypic. In addition, one species of the genus *Merizocera* Fage, 1912 is transferred to *Relictocera*: *Relictoceramus* (Deeleman-Reinhold, 1995), **comb. nov.** (♂♀). The types of the new species are deposited in the Institute of Zoology, Chinese Academy of Sciences (IZCAS) in Beijing.

## Introduction

The spider family Psilodercidae Machado, 1951 was previously considered to be a subfamily of Ochyroceratidae Fage, 1912 by Machado (1951), and this was supported by Deeleman-Reinhold (1995). Psilodercids were elevated to family rank by Wunderlich (2004, 2008). Recently, phylogenetic analyses suggest that Psilodercidae are the sister group to the lineage encompassing Sicariidae Keyserling, 1880 and Scytodidae Blackwall, 1864 (Shao and Li 2018).

Psilodercidae comprises 120 named species in 11 genera (WSC 2019; Li and Quan 2017). Of these species, half of them belong to the genus *Althepus* Thorell, 1898. The other genera, such as *Flexicrurum* Tong & Li, 2007, *Luzonacera* Li & Li, 2017, *Qiongocera* Li & Li, 2017, *Relictocera* Li & Li, 2017, *Sinoderces* Li & Li, 2017, and *Thaiderces* Li & Li, 2017, have only recently been described (Tong and Li 2007; Liu et al. 2017). These haplogyne spiders are small web-weavers that are diverse in Southeast Asia. In total, 36 psilodercid species are reported from Thailand, 28 from Indonesia, 13 from China, 8 from Malaysia, 6 from Myanmar, 5 from Philippines, 5 from Laos and 3 from Vietnam (WSC 2019). The majority of the species are local endemics (WSC 2019).

While studying material from China, Thailand, and Vietnam, we found four new species belonging to two genera: *Qiongocera* and *Relictocera*, previously thought to be monotypic (Liu et al. 2017). The goal of this paper is to provide descriptions of the new species as well as new updated diagnoses for the two genera.

## Materials and methods

Types are deposited in the Institute of Zoology, Chinese Academy of Sciences (IZCAS) in Beijing. All specimens collected were preserved and observed in a 95% ethanol solution. The specimens were measured and examined under a Leica M205C stereomicroscope, and further morphological details were observed using an Olympus BX41 compound microscope. The male palp was dissected from the left side of the spider for further examination. The carapace measurements include the clypeus (except for *Relictocera* sp. which has a distinct clypeus). The length and width ratios were measured according to the length of the cymbium (including the cymbial protrusion) to its width. The internal genitalia of the female and the male palp were dissected and immersed in lactic acid for digestion. An Olympus C7070 wide zoom digital camera (7.1 megapixels) mounted on an Olympus SZX12 stereomicroscope was used to take photos at different focal plans. The photos were assembled with the image stacking software Helicon Focus 6.7.1 to generate high quality photos before further editing with Adobe Photoshop CC 2014. Leg measurements are given as total length (femur, patella, tibia, metatarsus, and tarsus). Leg segments were measured from their retrolateral side. All measurements are given in millimetres (mm). Terminology follows Li et al. (2014), Tong and Li (2007), and Deeleman-Reinhold (1995).

## Taxonomy

### Family Psilodercidae Machado, 1951

#### Genus Qiongocera

Taxon classificationAnimaliaAraneaePsilodercidae

Li & Li, 2017

##### Type species.

**Figure 1.**
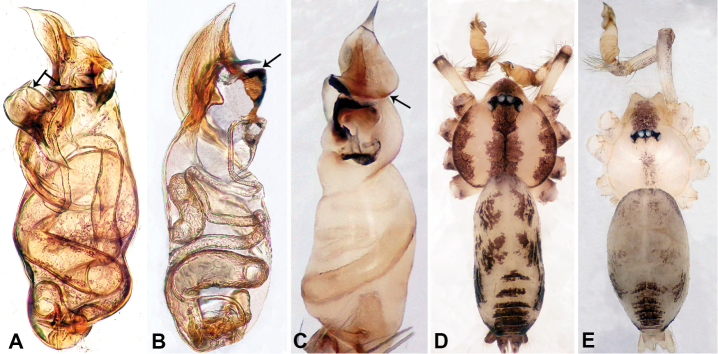
Bulb and habitus of *Qiongocerahongjunensis* (**A**, **D**) and *Q.luoxuan* sp. nov. (**B–C**, **E**) **A** bulb, prolateral **B** bulb, retrolateral **C** bulb, ventral **D–E** habitus, dorsal.

*Qiongocerahongjunensis* Li & Li, 2017 from China, Hainan Province. Details and figures of the type species as in Fig. 1A, D, and Liu et al. (2017): figs 5, 6.

##### Emended diagnosis.

*Qiongocera* resembles *Flexicrurum* but can be differentiated by the following combination of characters: 1) cymbium with a tilted protrusion (vs. cymbium with a strong lateral protrusion and with a small postero-lateral protrusion bearing a strong seta; 2) laminar apophysis half the width and length of bulb (vs. bulbal apophysis length equals and width, 15 times shorter than that of bulb in *Flexicrurum*); 3) simple process on the distal end of bulb (vs. a complex processes with more than one extension in *Flexicrurum*); and 4) a short embolus (vs. a long embolus in *Flexicrurum*).

##### Composition.

*Qiongocerahongjunensis* (the type species) and *Qiongoceraluoxuan* Li & Li, sp. nov.

##### Distribution.

Hainan Province, China.

#### Key to species of *Qiongocera* (males only)

**Table:** 

1	Bulb with laminar apophysis on distal part and with 2 unequal parts of a plier-like processes (Fig. 1A); body dark brown (Fig. 1D)	*** Q. hongjunensis ***
–	Bulb with a distinct helical laminar apophysis extending from the distal half (Fig. 1C) and with a triangular process (Fig. 1B); body pale (Fig. 1E)	***Q.luoxuan* sp. nov.**

##### Qiongocera luoxuan

Taxon classificationAnimaliaAraneaePsilodercidae

Li & Li sp. nov.

http://zoobank.org/76C26986-8691-4315-B226-3E8FA56A11DA

Figs 1B, C, E , 4 , 5 , 12A , 13

###### Types.

**Holotype**: ♂ (IZCAS), China, Hainan Province, Dongfang City, Donghe Town, Yalong Village, Yalong-Huangxian Cave, 18°58.752'N, 108°53.308'E, 264 m, 15.XII.2014, Zhao Q. and Shao L. **Paratypes**: 1♂1♀ (IZCAS), same data as holotype.

###### Etymology.

The species name is a noun in apposition derived from the Chinese pinyin “luόxuán” (helical) and refers to the helical shape of the bulb in the distal half.

###### Diagnosis.

Males of *Q.luoxuan* sp. nov. can be distinguished from *Q.hongjunensis* by the triangular process on the bulb (Fig. 5B) (vs. a plier-like process with 2 unequal parts in *Q.hongjunensis*); the distal half of the bulb helical (Fig. 5C, D) (vs. a rather simple pyriform bulb in *Q.hongjunensis*); females can be distinguished by the pair of thin, complex branches of the spermathecae that are convexly curved (Fig. 4A) (vs. a pair of bulging round spermathecae in *Q.hongjunensis*); the coloration and patterns of both male and female are relatively indistinct (Fig. 4C–E) (vs. a distinct dark brown coloration and pattern in *Q.hongjunensis*).

###### Description.

**Male** (Holotype). Total length 2.65; carapace 1.09 long, 1.13 wide; abdomen 1.56 long, 0.78 wide. Carapace round and pale yellow with a faint longitudinal brown patch medially (Fig. 4C). Fovea shallow and brown. Anterior part of the thoracic region distinctly elevated. Chelicerae yellow. Cheliceral promargin with lamina bearing 3 triangular extensions, retromargin with 2 small teeth (Fig. 12A). Clypeus slanting, dark brown with two pale areas laterally. Endites pale yellow. Labium slanting, light brown. Sternum circular with brown complex pattern delimiting two pale oval areas anteriorly and posteriorly. Abdomen dorsum with several dark horizontal stripes concentrated posteriorly, venter with dark curves concentrated at the edges. Legs uniformly brown; measurements: I 18.75 (5.45, 0.40, 5.77, 5.13, 2.00), II 14.02 (4.00, 0.40, 4.49, 3.53, 1.60), III missing, IV missing. Palp (Fig. 5A–D): femur slender, 5 times longer than patella; patella angled ventrally; tibia pale, 2 times shorter than femur, suffused with tiny sepia patches, strongly deflected prolaterally; cymbium pale, darker distally, with dorsal margin curved basally, with short, pale distal protrusion, conical in lateral view and directed at 45 °; length/width ratio = 2.0; bulb light yellow, proximal half roughly cylindrical, distal half twisted, forming 3 helical coils tapered apically, ending at a pointed laminar apophysis; triangular process adjacent to embolus short; embolus thin, sharp and darkened, extending from distal part of the bulb (Fig. 5B).

**Female** (Paratype). General features and coloration similar to that of male (Fig. 4D–E). Measurements: total length 2.60; carapace 1.00 long, 1.00 wide; abdomen 1.60 long, 0.80 wide. Leg measurements: I 12.85 (3.50, 0.40, 3.75, 3.40, 1.80), II missing, III 7.16 (2.00, 0.31, 2.19, 1.72, 0.94), IV 10.39 (3.20, 0.30, 3.30, 2.50, 1.09). Internal genitalia: one pair of sclerotized ovoid spermathecae surrounded by a pair of thin ducts with complex branches curving convexly, posteriorly with horizontal bar bearing droplet-shaped ducts (Fig. 4A).

###### Distribution.

Known only from the type locality (Fig. 13).

##### Genus Relictocera

Taxon classificationAnimaliaAraneaePsilodercidae

Li & Li, 2017

###### Type species.

**Figure 2.**
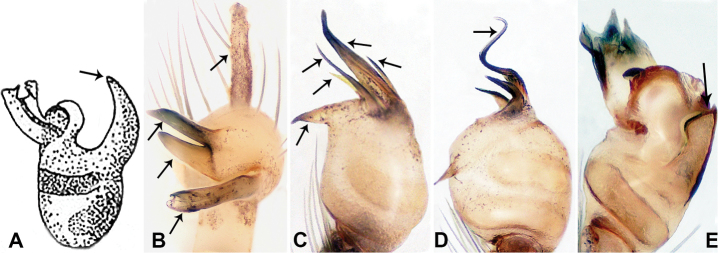
Bulbs of *Relictoceramus* (**A**) *R.qiyi* (**B**) *R.wugen* sp. nov. (**C**) *R.sigen* sp. nov. (**D**) and *R.qianzi* sp. nov. **(E). A** retrolateral view; **B–E** ventral views.

**Figure 3.**
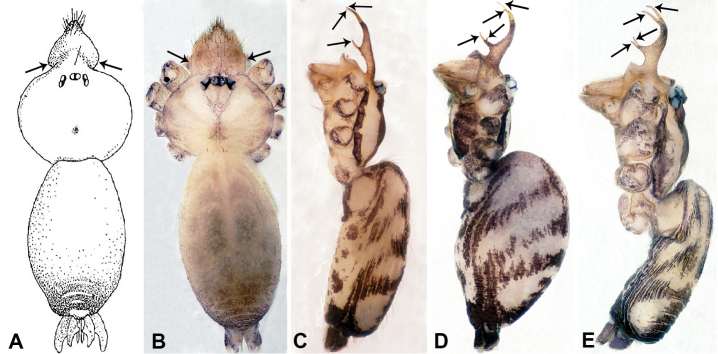
Male habitus of *Relictoceramus* (**A**) *R.qianzi* sp. nov. (**B**) *R.qiyi* (**C**) *R.wugen* sp. nov. (**D**) *R.sigen* sp. nov. (**E**). **A–B** dorsal views; **C–E** lateral views.

*Relictoceraqiyi* Li & Li, 2017 from Vietnam, Thua Thien Hue Province. Details and figures of type species as shown in Figs 2B, 3C and Liu et al. (2017): figs 7, 8.

**Figure 4.**
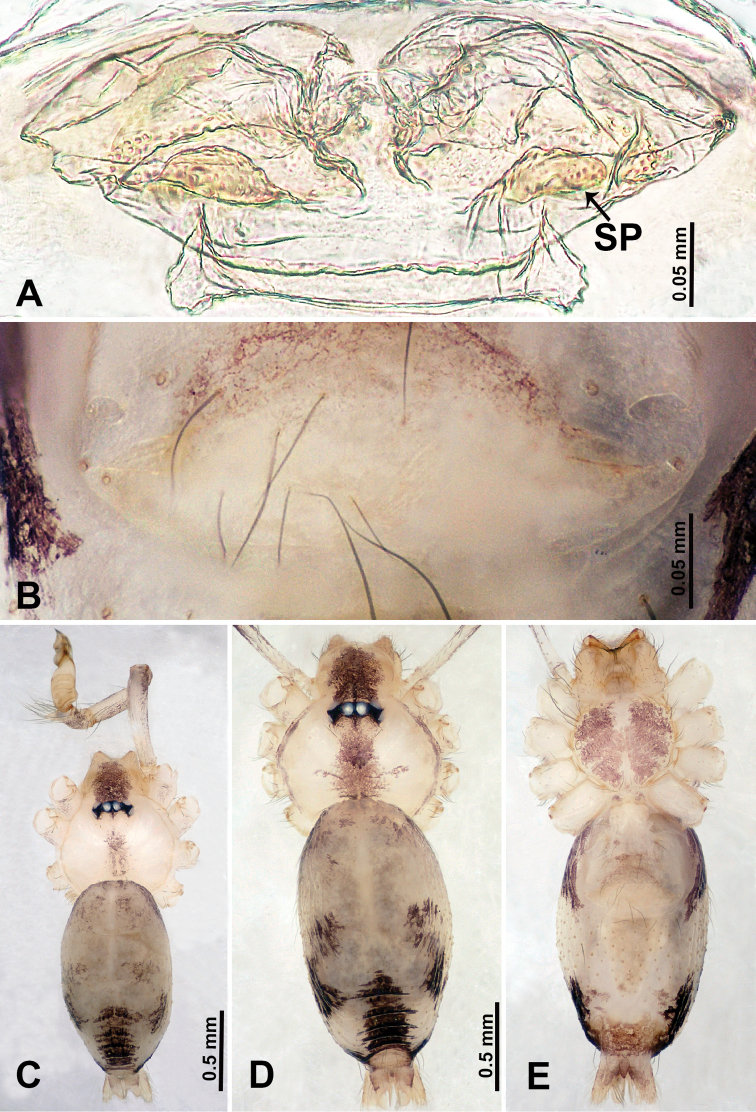
*Qiongoceraluoxuan* sp. nov., male holotype and female paratype **A** internal genitalia, dorsal view **B** female epigastric area, ventral view **C** male habitus, dorsal view **D** female habitus, dorsal view **E** female habitus, ventral view. Abbreviation: **SP** = spermatheca.

**Figure 5.**
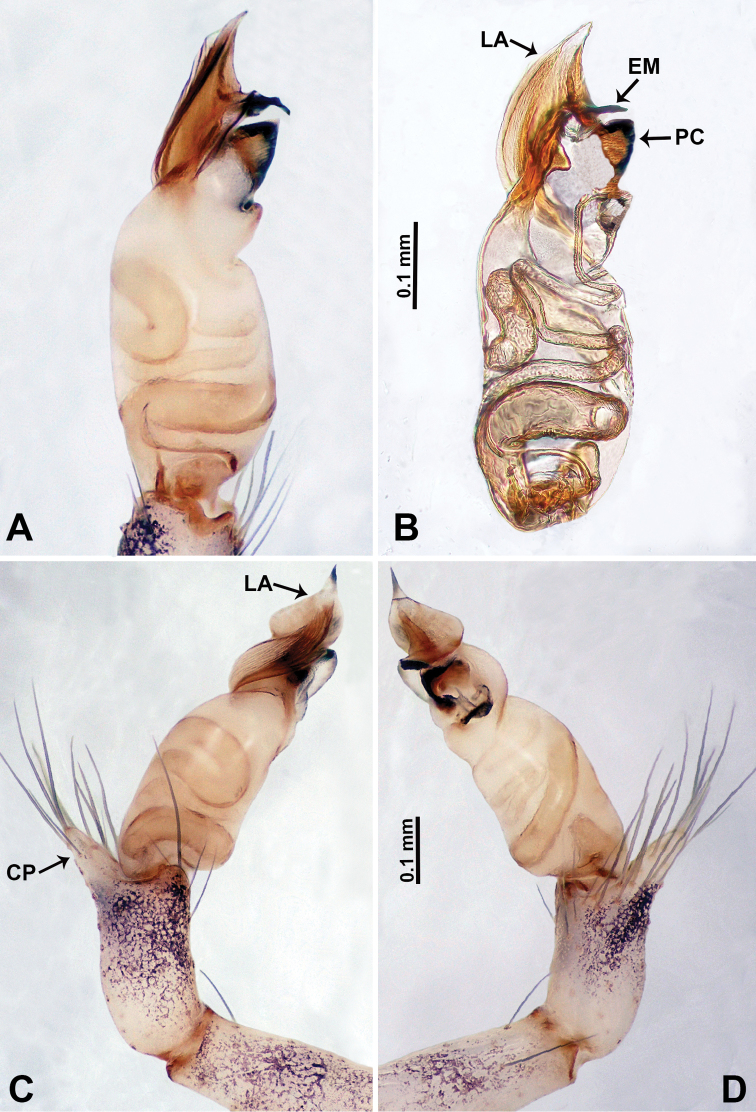
*Qiongoceraluoxuan* sp. nov., male holotype **A** palp, ventral view **B** palpal bulb, ventral view **C** palp, prolateral view **D** palp, retrolateral view. Abbreviations: **CP** = cymbial protrusion, **EM** = embolus, **LA** = laminal apophysis, **PC** = process of bulb.

###### Emended diagnosis.

*Relictocera* can be distinguished from *Luzonacera* by the following combination of characters: 1) bulb with numerous appendages (vs. bulb without appendages); 2) male with clypeal projection (vs. male without clypeal projection); 3) chelicera without promarginal teeth (vs. chelicera with 1 promarginal tooth); and 4) cheliceral promargin lamina with 3 triangular extensions (vs. lamina with 2 triangular extensions).

###### Composition.

*Relictoceraqiyi* (the type species), *R.sigen* Li & Li, sp. nov., *R.wugen* Li & Li, sp. nov., *R.qianzi* Li & Li, sp. nov., and *R.mus* (Deeleman-Reinhold, 1995).

###### Distribution.

Vietnam and Thailand.

#### Key to species of *Relictocera* (males only)

**Table:** 

1	Bulb with pincer-like appendages; clypeus with a hairy snout-like projection; patella 2 times wider than cymbium	**2**
–	Bulb with tentacle-like appendages; clypeus with a furcate projection; patella and cymbium equally wide	**3**
2	Appendages with pointed tips (Fig. 2A); clypeus with rounded base (Fig. 3A); carapace without pattern (Fig. 3A)	*** R. mus ***
–	Appendages with blunt tips (Fig. 2E); clypeus with a straight base (Fig. 3B); carapace with distinct pattern (Fig. 3B)	***R.qianzi* sp. nov.**
3	Embolus straight	**4**
–	Embolus spiralled (Fig. 2D)	***R.sigen* sp. nov.**
4	Bulb with 5 appendages (including embolus) (Fig. 2C); appendages of different length; clypeus with a quadrifurcate projection (Fig. 3D)	***R.wugen* sp. nov.**
–	Bulb with 4 appendages (including embolus) (Fig. 2B); appendages (except embolus) almost equal in length; clypeus with a trifurcate projection (Fig. 3C)	*** R. qiyi ***

##### Relictocera wugen

Taxon classificationAnimaliaAraneaePsilodercidae

Li & Li sp. nov.

http://zoobank.org/0C9B73EC-29BF-44EC-9178-2E06E476D3CF

**Figure 6.**
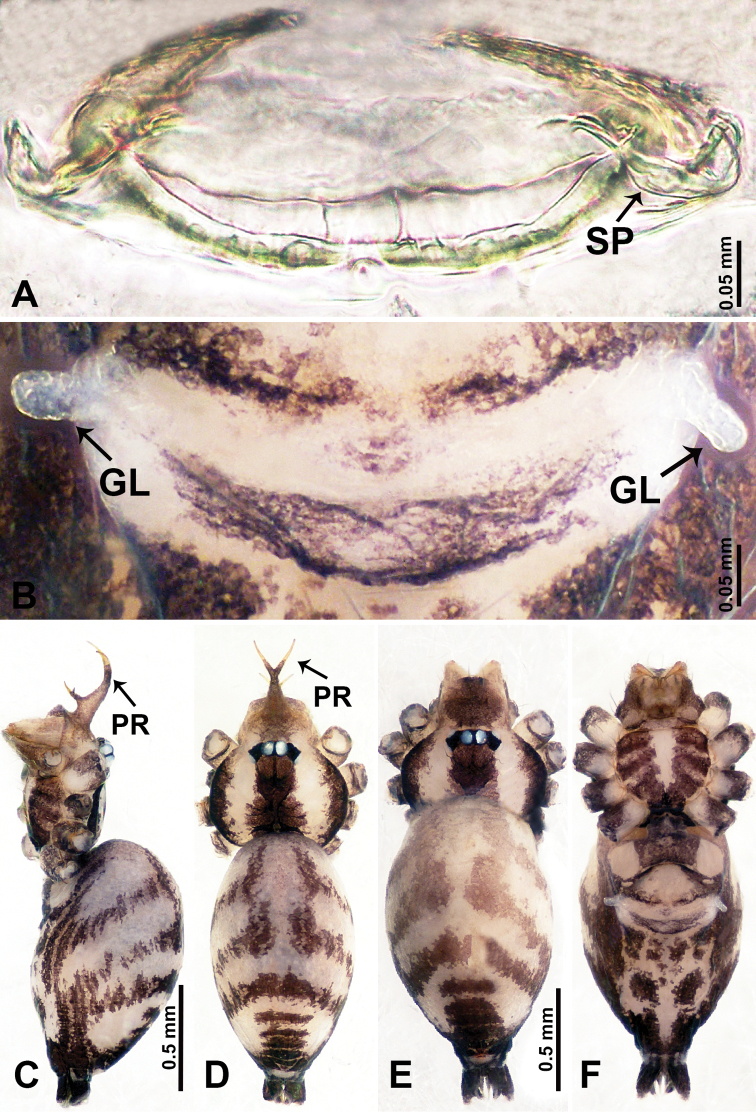
*Relictocerawugen* sp. nov., male holotype and female paratype **A** internal genitalia, dorsal view **B** female epigastric area, ventral view **C** male habitus, retrolateral view **D** male habitus, dorsal view **E** female habitus, dorsal view **F** female habitus, ventral view. Abbreviations: **GL** = genitalic lobe, **PR** = clypeal protrusion, **SP** = spermatheca.

**Figure 7.**
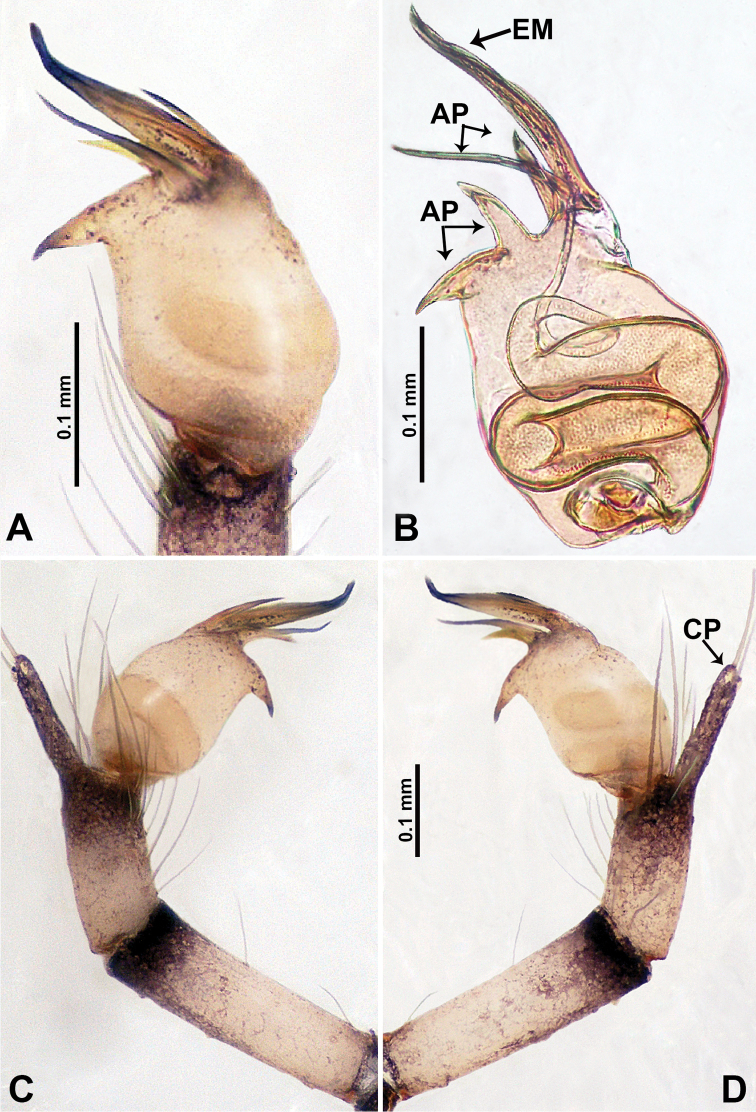
*Relictocerawugen* sp. nov., male holotype **A** palp, ventral view **B** palpal bulb, ventral view **C** palp, prolateral view **D** palp, retrolateral view. Abbreviations: **AP** = appendage of bulb, **CP** = cymbial protrusion, **EM** = embolus.

Figs 2C , 3D , 6 , 7 , 12B , 13

###### Types.

**Holotype**: ♂ (IZCAS), Vietnam, Quang Binh Province, Phong Nha-Ke Bang National Park, outside of Botanical Garden, 17°32.895'N, 106°17.830'E, 261 m, 23.VIII.2015, Zhao Q., Li Y. & Chen Z. **Paratypes**: 1♂1♀ (IZCAS), same data as holotype.

###### Etymology.

The species name is a noun in apposition derived from the Chinese pinyin “wugen” (“five stripes”) and refers to the five appendages on the male bulb, including the embolus.

###### Diagnosis.

Males of *R.wugen* sp. nov. can be distinguished from those of *R.sigen* sp. nov. by the nearly straight embolus (Fig. 7B) vs. a spiralled embolus Fig. 9B; *R.wugen* sp. nov. has 4 distinct and relatively long bulbal appendages (Fig. 7B) vs. 3 relatively short bulbal appendages (Fig. 9B); pyriform bulb (Fig. 7B) vs. globular bulb (Fig. 9B); carapace wider than long vs. longer than wide; females can be distinguished from *R.qianzi* sp n. by a pair of slender and elongated spermathecae that curve upward vs. ovoid spermathecae flanked laterally by a pair of translucent ducts (Fig. 6A).

###### Description.

**Male** (Holotype). Total length 1.70; carapace 0.70 long, 0.63 wide; abdomen 1.00 long, 0.69 wide. Carapace round, pale yellow, with 3 longitudinal dark brown bands; the middle band 2 times wider than the lateral band (Fig. 6D). Chelicerae yellow (Fig. 12B). Clypeus light brown, with a long, quadrifurcate medial projection (Fig. 6C, D). Endites brown. Labium brown with a pair of pale spots. Sternum with longitudinal white median band and pair of dark brown lateral stripes. Abdomen dorsally with longitudinal white band anteriorly and with 3 pairs of dark brown lateral stripes, posterior part with longitudinal dark brown stripes, anterior of ventrum with pair of circular pale lateral patches, posterior with dark brown lateral area and several longitudinal irregular dark brown spots (Fig. 6C, D). Legs uniformly brown; measurements: I 10.75 (2.75, 0.25, 3.25, 3.25, 1.25), II 7.50 (2.24, 0.25, 2.13, 2.13, 0.75), III 5.40 (1.60, 0.20, 1.50, 1.50, 0.60), IV missing. Palp (Fig. 7A–D): femur 5 times longer than patella; patella not swollen; tibia pale, 2 times shorter than femur, with dark distal ring; cymbium pale, distal half darker than proximal, with long and thin dark distal protrusion directed forward; length/width ratio = 3.13; bulb light yellow, oval, with 4 appendages; embolus straight, flattened, tapering apically; appendages (except embolus) differ in length, merging separately at distal part of bulb and adjacent to embolus (Fig. 7B).

**Female** (Paratype). General features and coloration similar to those of male except for the absence of a quadrifurcate clypeal projection (Fig. 6E, F). Measurements: total length 2.50; carapace 0.75 long, 0.80 wide; abdomen 1.75 long, 0.80 wide. Leg measurements: I 6.54 (2.00, 0.16, 2.19, 1.19, 1.00), II 5.56 (1.56, 0.25, 1.50, 1.50, 0.75), III 3.90 (1.09, 0.16, 1.09, 1.09, 0.47), IV 6.25 (1.75, 0.25, 1.75, 1.75, 0.75). Epigastric furrow (slit) with pair of translucent lateral lobes (Fig. 6B). Internal genitalia: one pair of slender and elongated spermathecae curving anteriorly with pointed tips, bases separated by 3 times the width of the spacing of the of tips. (Fig. 6A).

###### Distribution.

Known only from the type locality (Fig. 13).

##### Relictocera sigen

Taxon classificationAnimaliaAraneaePsilodercidae

Li & Li sp. nov.

http://zoobank.org/6462B938-880A-4C6A-BDFE-64058181721C

**Figure 8.**
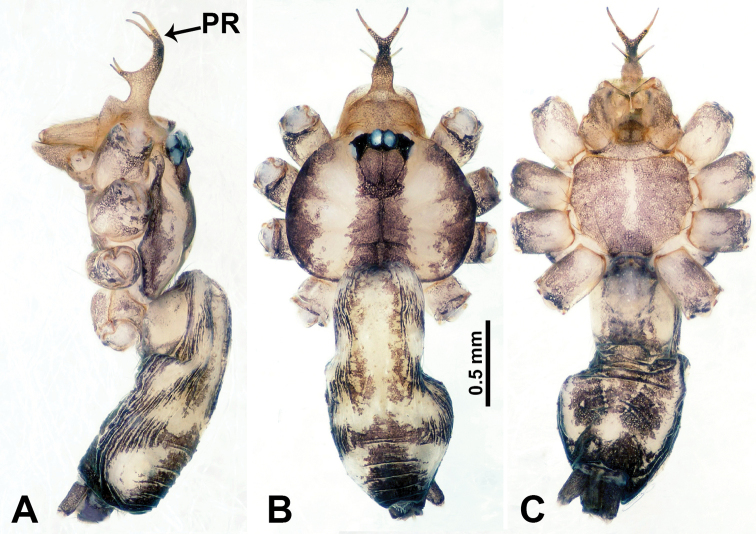
*Relictocerasigen* sp. nov., male holotype **A** male habitus, retrolateral view **B** male habitus, dorsal view **C** male habitus, ventral view. Abbreviation: **PR** = clypeal projection.

**Figure 9.**
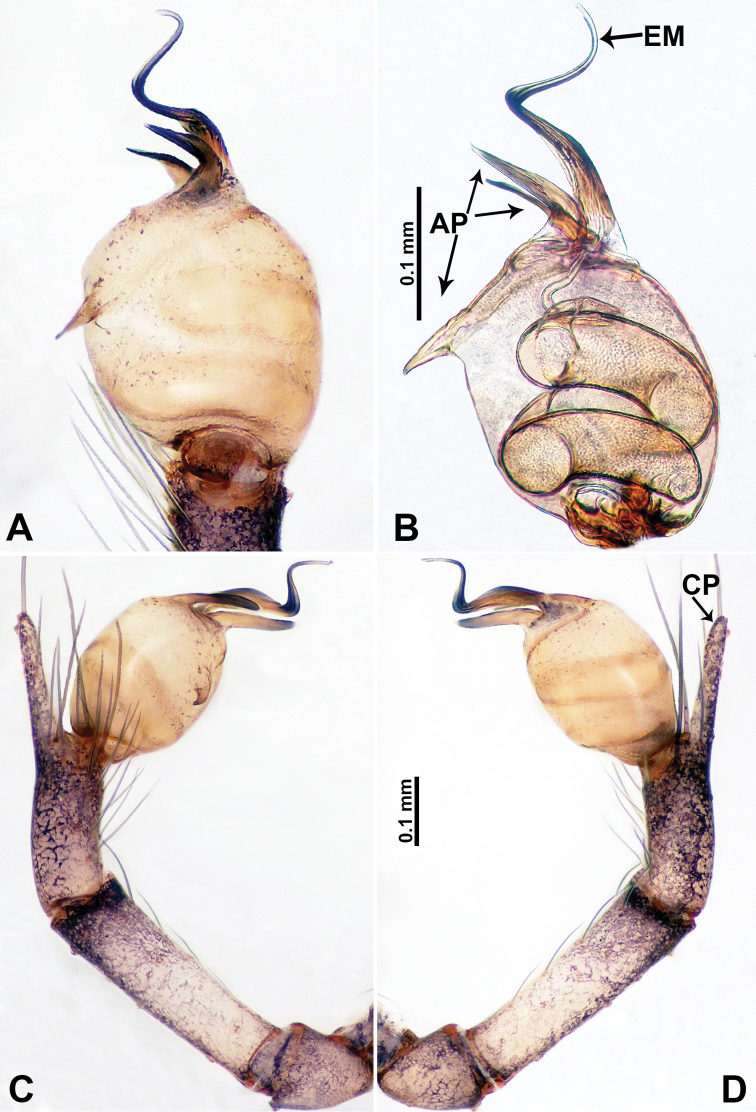
*Relictocerasigen* sp. nov., male holotype **A** palp, ventral view **B** palpal bulb, ventral view **C** palp, prolateral view **D** palp, retrolateral view. Abbreviations: **AP** = appendage of bulb, **CP** = cymbial protrusion, **EM** = embolus.

Figs 2D , 3E , 8 , 9 , 12C , 13

###### Types.

**Holotype**: ♂ (IZCAS), Vietnam, Ninh Binh Province, Cuc Phuong National Park, Palace Cave, 20°21.350'N, 105°36.282'E, 523 m, 19.VIII.2015, Zhao Q., Li Y. & Chen Z.

###### Etymology.

The species name is a noun in apposition derived from the Chinese pinyin “sigen” (“four strips”) and refers to the four appendages on the male bulb, including the embolus.

###### Diagnosis.

See diagnosis for *R.wugen* sp. nov.

###### Description.

**Male** (Holotype). Total length 2.28; carapace 1.00 long, 1.20 wide; abdomen 1.28 long, 0.64 wide. Carapace round, yellow, with 3 longitudinal dark brown bands; the middle band 2 times wider than the lateral band (Fig. 8B). Chelicerae yellow (Fig. 12C). Clypeus pale brown, with quadrifurcate medial projection (Fig. 8A, B). Endites pale brown. Labium brown with 2 large pale spots (Fig. 8C). Sternum brown with median longitudinal pale band not reaching the posterior margin (Fig. 8C). Abdomen dorsally with anterior longitudinal pale-yellow band and pair of lateral dark brown stripes, posteriorly with longitudinal dark brown stripes, venter pale anteriorly, posterior part with scattered dark brown patches. Legs uniformly brown; measurements: I (3.51, 0.40, 5.77, 6.73, missing), II 12.70 (3.53, 0.32, 3.85, 4.00, 1.00), III 9.40 (2.60, 0.40, 2.80, 2.80, 0.80), IV 15.39 (4.49, 0.32, 4.49, 4.81, 1.28). Palp (Fig. 9A–D): femur 5 times longer than patella; patella not swollen; tibia pale, 2 times shorter than femur, dark distally; cymbium almost completely dark, with long and thin distal protrusion directed forward, slightly oblique; length/width ratio= 3.33; bulb light yellow, globose, with 3 appendages (except embolus); embolus coiled, tapering apically; trifurcate appendages almost equal in length but distinctly shorter than embolus, emerging separately at distal part of bulb, except for one prolateral appendage directed perpendicular to other appendages (Fig. 9B).

**Figure 10.**
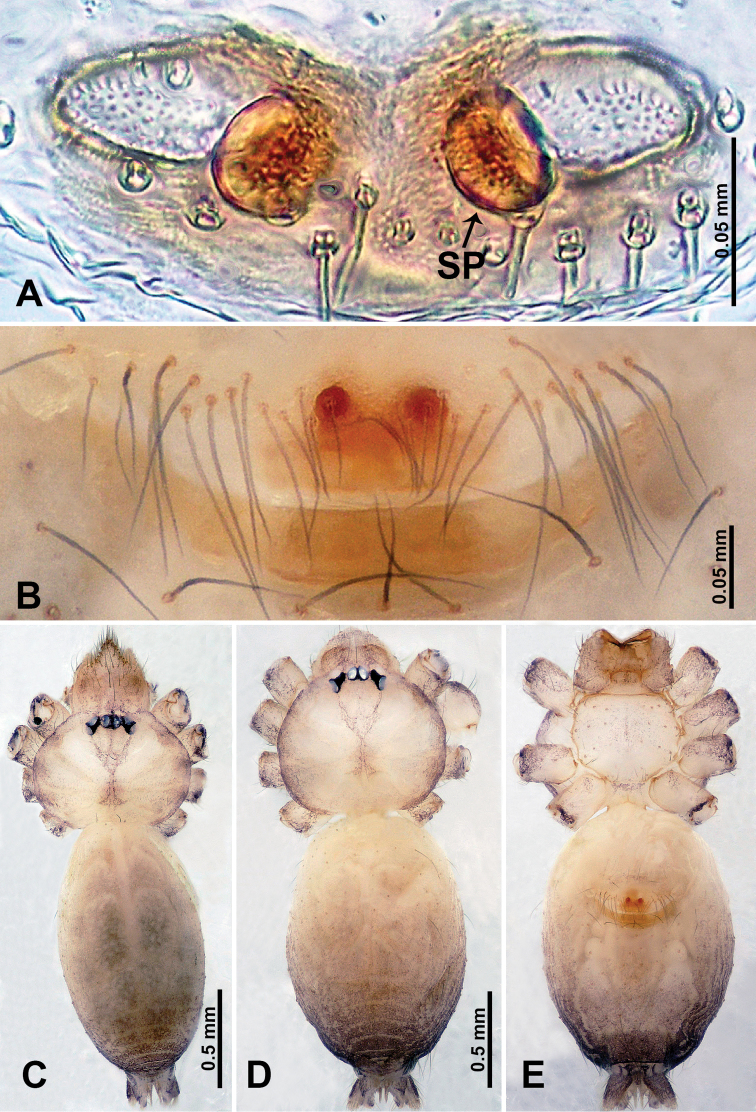
*Relictoceraqianzi* sp. nov., male holotype and female paratype **A** internal genitalia, dorsal view **B** female epigastric area, ventral view **C** male habitus, dorsal view **D** female habitus, dorsal view **E** female habitus, ventral view. Abbreviation: **SP** = spermatheca.

**Figure 11.**
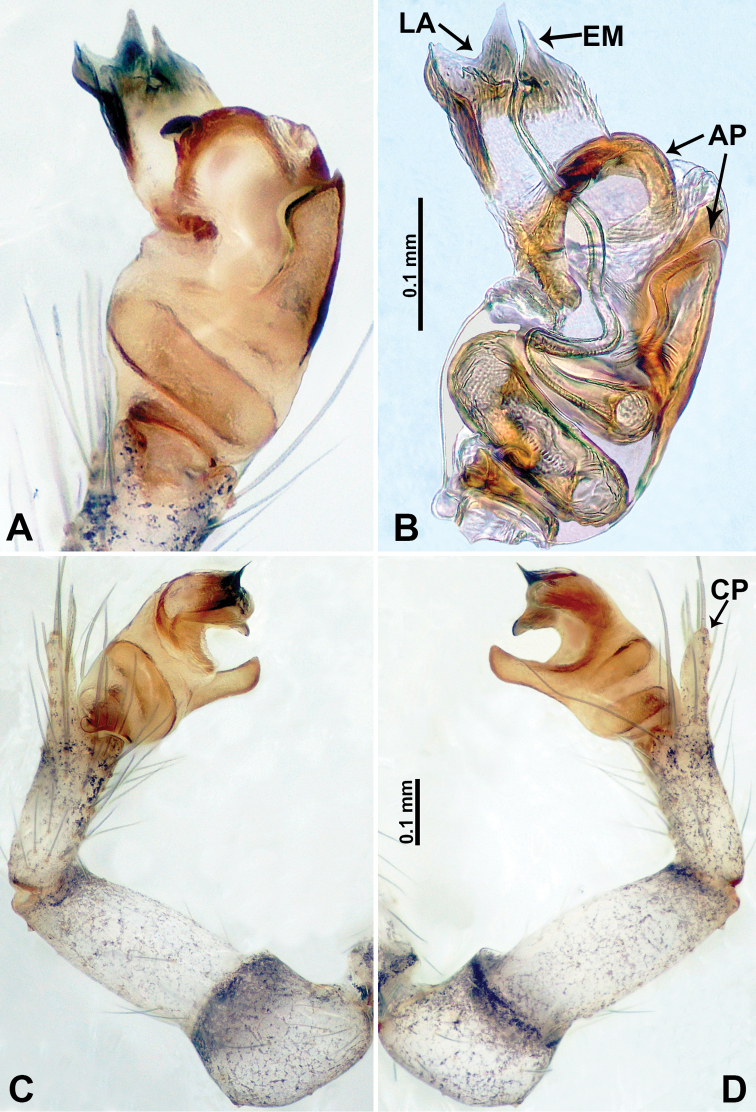
*Relictoceraqianzi* sp. nov., male holotype **A** palp, ventral view **B** palpal bulb, ventral view **C** palp, prolateral view **D** palp, retrolateral view. Abbreviations: **AP** = appendage, **CP** = cymbial protrusion, **EM** = embolus, **LA** = laminar apophysis.

**Figure 12.**
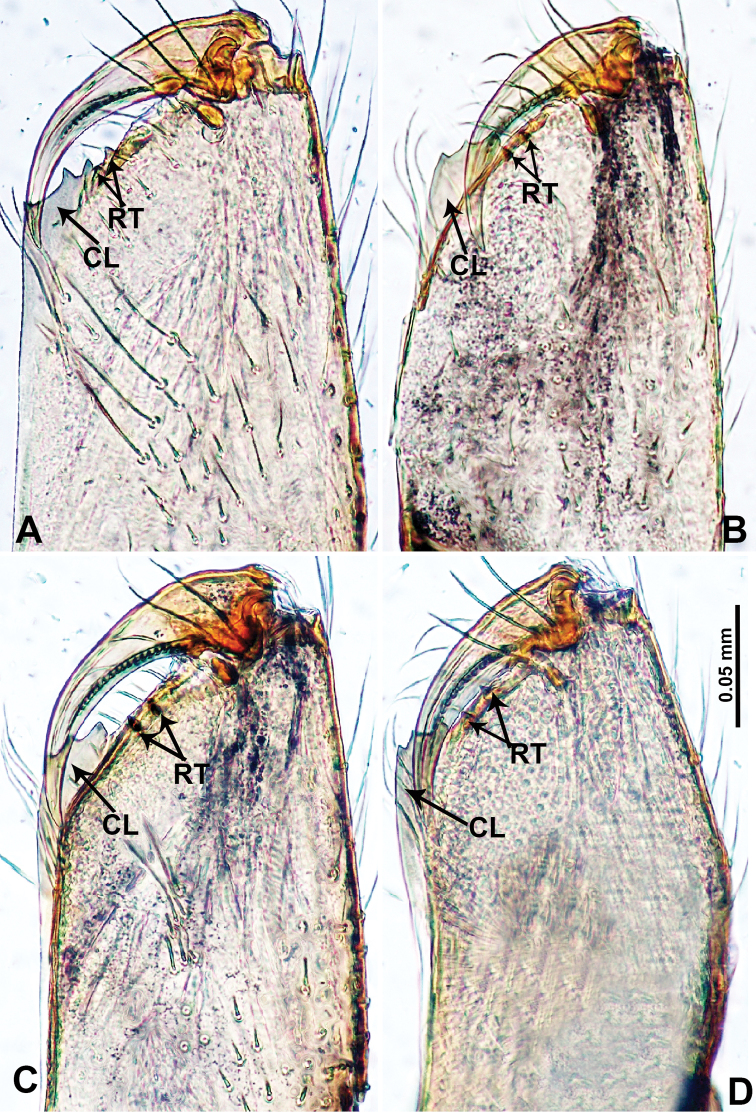
Cheliceral retromargin **A***Qiongoceraluoxuan* sp. nov. **B***Relictocerawugen* sp. nov. **C***R.sigen* sp. nov. **D***R.qianzi* sp. nov. Abbreviations: **CL** = cheliceral lamina, **RT** = retromarginal teeth.

###### Female.

Unknown.

###### Distribution.

**Figure 13.**
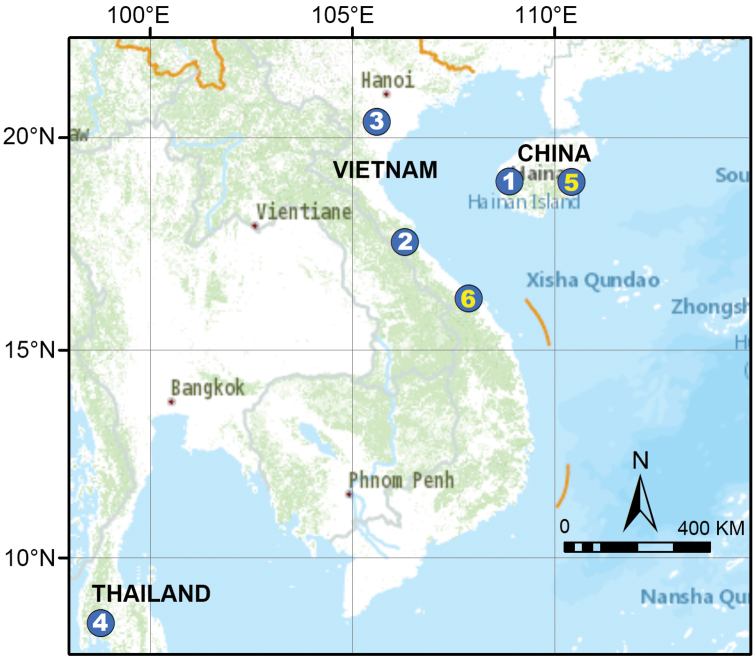
Distribution of *Qiongocera* and *Relictocera* species in China (Hainan), Vietnam, and Thailand. White numbers indicate new species and yellow numbers indicate previously known species. **1***Qiongoceraluoxuan* sp. nov. **2***Relictocerawugen* sp. nov. **3***R.sigen* sp. nov. **4***R.qianzi* sp. nov. **5***Qiongocerahongjunensis***6***Relictoceraqiyi*.

Known only from the type locality (Fig. 13).

##### Relictocera qianzi

Taxon classificationAnimaliaAraneaePsilodercidae

Li & Li sp. nov.

http://zoobank.org/02E7AC89-2B89-4108-B450-F9DBAD1D30F4

Figs 2E , 3B , 10 , 11 , 12D , 13

###### Types.

**Holotype**: ♂ (IZCAS), Thailand, Krabi Province, Ao Luk District, Petch Cave, 8°23.578'N, 98°46.437'E, 56 m, 11.X.2015, Zhao Q., Zhou G. & Chen Z. **Paratypes**: 1♂1♀ (IZCAS), same data as holotype.

###### Etymology.

The species name is a noun in apposition derived from the Chinese pinyin “qiánzĭ” (pincer) and refers to the unique structure of the bulb appendages which are similar to the gripping jaws of a pincer (Fig. 11C, D).

###### Diagnosis.

Males of *R.qianzi* sp. nov. can be distinguished from all other congeners by the bulb bearing two unequal parts, a strongly swollen palpal patella, and a partly swollen tibia (Fig. 11C–D). Males can be easily distinguished from *R.mus* by the blunt tips of the bulbal appendages (vs. sharply pointed tips); a hairy snout-liked clypeus with a straight base (vs. a spade-shaped clypeus with a distinct rounded base in *R.mus*); carapace with trident pattern medially (vs. absence of pattern); the female can be distinguished from other species by the pair of ovoid spermathecae flanked laterally with a pair of translucent ducts (vs. a pair of horizontal ducts bearing round spermathecae).

###### Description.

**Male** (Holotype). Total length 2.70; carapace 1.10 long, 1.10 wide; abdomen 1.60 long, 0.90 wide. Carapace round and pale yellow, medially with light brown trident pattern, laterally slightly darker (Fig. 10C). Chelicerae yellow (Fig. 12D). Clypeus brown, with hairy snout-like projection. Endites pale brown. Labium brown, delimiting 2 oval paler areas. Sternum yellow. Abdomen with dorsal longitudinal pale band, with shaded lateral patches, posterior part with a few dark brown stripes, anterior part of ventrum pale, posterior part with dark brown shading on the margin. Legs uniformly brown; measurements: I missing, II missing, III 10.48 (3.00, 0.31, 2.97, 3.20, 1.00), IV missing. Palp (Fig. 11A–D): femur slender, 2 times longer than patella; patella strongly swollen, as long as wide, 2 times wider than cymbium; tibia 2/3 the length of femur, basally swollen (length/width ratio = 3.0); cymbium pale, slightly yellowish distally, with distal protrusion pale, slightly curved upward; length/width ratio = 3.00; bulb brown, bifurcate; laminar apophysis branched; embolus located distally, adjacent to laminar apophysis, ribbon-like with a protruding tip and a pair of irregular pincer-like appendages adjacent to the embolus (Fig. 11C, D).

###### Female.

General features and coloration similar to those of the male (Fig. 10D, E). Measurements: total length 2.46; carapace 0.90 long, 1.09 wide; abdomen 1.56 long, 0.94 wide. Leg measurements: I 16.73 (4.00, 0.40, 4.81, 5.77, 1.75), II 11.68 (3.20, 0.32, 3.21, 3.75, 1.20), III missing, IV missing. Epigastric area with two small, round red spots medially, anterior to epigastric furrow; posterior margin of epigastric slit thick, slightly sclerotized (Fig. 10B, E). Internal genitalia: one pair of sclerotized ovoid spermathecae flanked laterally by 2 larger translucent ducts, spermathecae separated by one spermatheca diameter (Fig. 10A).

###### Distribution.

Known only from the type locality (Fig. 13).

##### Relictocera mus

Taxon classificationAnimaliaAraneaePsilodercidae

(Deeleman-Reinhold, 1995) comb. nov.

Figs 2A , 3A

Merizocera mus Deeleman-Reinhold 1995: 42, figs 94–103 (♂♀)

###### Diagnosis.

Diagnostic features are discussed under *R.qianzi* sp. nov.

###### Description.

Described by Deeleman-Reinhold (1995).

###### Distribution.

Thailand.

###### Remarks.

Although we did not examine the type material of *Merizoceramus*, figures of this species in Deeleman-Reinhold (1995) are congruent with the features of the *Relictocera*.

## Supplementary Material

XML Treatment for Qiongocera

XML Treatment for Qiongocera luoxuan

XML Treatment for Relictocera

XML Treatment for Relictocera wugen

XML Treatment for Relictocera sigen

XML Treatment for Relictocera qianzi

XML Treatment for Relictocera mus
